# Development, Characterization, and In Vitro Evaluation of Resveratrol-Loaded Poly-(ε-caprolactone) Microcapsules Prepared by Ultrasonic Atomization for Intra-Articular Administration

**DOI:** 10.3390/pharmaceutics11060249

**Published:** 2019-05-28

**Authors:** Asteria Luzardo-Álvarez, Iván Lamela-Gómez, Francisco Otero-Espinar, José Blanco-Méndez

**Affiliations:** 1 Department of Pharmacology, Pharmacy and Pharmaceutical Technology, School of Sciences, Campus de Lugo, University of Santiago de Compostela, 27002 Lugo, Spain; ivan.lamela@usc.es (I.L.-G.); jose.blanco.mendez@usc.es (J.B.-M.); 2 Department of Pharmacology, Pharmacy and Pharmaceutical Technology, School of Pharmacy, Campus de Santiago de Compostela, University of Santiago de Compostela, 14875 Santiago de Compostela, Spain; francisco.otero@usc.es

**Keywords:** drug delivery system, microcapsules, photodegradation, ultrasound, antioxidant, site-specific delivery, resveratrol

## Abstract

Intra-articular administration of drugs to the joint in the treatment of joint disease has the potential to minimize the systemic bioavailability and the usual side-effects associated with oral drug administration. In this work, a drug delivery system is proposed to achieve an anti-inflammatory local effect using resveratrol (RSV). This study aims to develop microcapsules made of poly-(ε-caprolactone) (PCL) by ultrasonic atomization to preserve the antioxidant activity of RSV, to prevent its degradation and to suppress the inflammatory response in activated RAW 264.7 macrophages. An experimental design was performed to build a mathematical model that could estimate the effect of nozzle power and polymer concentration on particle size and encapsulation efficiency. RSV-loaded microcapsules showed adequate morphology, particle size, and loading efficiency properties. RSV formulations exhibited negligible cytotoxicity and an efficient amelioration of inflammatory responses, in terms of Nitric Oxide (NO), ROS (Reactive Oxygen Species), and lipid peroxidation in macrophages. Thus, RSV-loaded microcapsules merit consideration as a drug delivery system suitable for intra-articular administration in inflammatory disorders affecting the joint.

## 1. Introduction

**Figure 1:**
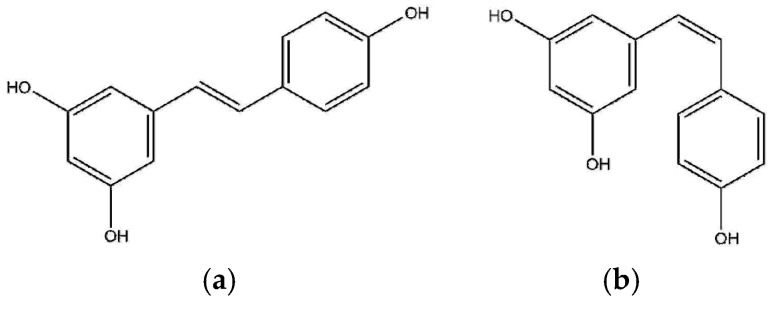
Structure of (**a**) *trans*-resveratrol (3,5,4′-trihydroxystilbene) and (**b**) *cis*-resveratrol.

*Trans*-resveratrol (3,5,4′-trihydroxystilbene; RSV) is a phytoalexin that has been found as a secondary metabolite in numerous vascular plants (Figure 1). As a polyphenolic compound, RSV has in common with other phenolic compounds the presence of one or more benzene rings that support one or more hydroxyl groups. Specifically, the groups 3- and 5-OH, in ring A, and the double link with 4-oxo function, in ring C, provide the antioxidant capacity of RSV. The phenomenon of oxidative stress and, as a consequence, the damage to cells and tissues by free radicals has been demonstrated to play a critical role in a wide number of pathologies including, among others, rheumatoid arthritis, where the oxidative stress is even considered a pathogenic marker of the disease [1,2]. The antioxidant power of RSV has been well documented and supports RSV as a promising molecule to prevent free radical damage, which might be responsible for the anti-inflammatory effects found in vitro [3] and in vivo studies [4]. Nonetheless, the antioxidant properties of RSV are limited by its poor oral bioavailability and short half-life, its poor solubility in water (0.03 mg/mL), and its chemical instability. Specifically, it is extremely sensitive against UV light [5]. In fact, RSV in dissolution and exposed to wavelengths of 254 or 366 nm rapidly isomerizes from *trans*-RSV (active isomeric form) to *cis*-RSV, to the extent of 91% after 2 h [6,7]. Additionally, RSV is unstable in neutral and high pH buffer (including physiological pH), even though it is protected from light.

From the point of view of a biomedical application, a solid dosage form that could sustain RSV release and remain in contact with the inflamed area for extended periods of time is ideal. If RSV stability may be improved from minutes to hours, then the local bioavailability would be enhanced and, in consequence, its biological effect as well [8]. Considering the low stability and solubility of *trans*-RSV, an encapsulation method of RSV in a biocompatible material coating is required to maximize the drug concentration and release at the inflammatory site of the organism. Within this context, microcapsules are potentially useful because of the ease of administration in local sites, by injection (sphericity), and the ability to increase the residence time within the joint. Therefore, it may diminish the dosage frequency to the patient and, most of all, prevent the biodegradation of RSV against pH and UV light. In the view of this biomedical application, the mean size of microcapsules is critical to prolong the residence time within the joint. With this in mind, a prolonged intra-articular delivery could be achieved by the slow degradation of the drug delivery system. This strategy could serve to prolong the retention at the site of injection and, as a consequence, the reduction of the dosage frequency and patient compliance.

Macrophages play a critical role in inflammatory reactions on diseases affecting the synovial joints, such as rheumatoid arthritis. It is an autoimmune disease of unknown etiology, which is accompanied by long-term inflammation and affects predominantly the articular tissue. The infiltration and the activation of macrophages in the synovial inflammatory cavities contribute to the degradation of cartilage and bone by the secretion of a wide number of pro-inflammatory factors (tumor necrosis factor-α, interleukin-1, nitric oxide production, cyclooxygenase mediators, or interleukin-8). Additionally, in the inflamed joint, activated macrophages can generate reactive oxygen species that are also involved in the degradation of the surrounding tissues [9]. Oxidative stress is an important contributing factor in arthritis because ROS promote cartilage destruction and induce chronic inflammation. In addition, lipid radicals, as products coming from lipid peroxidation, act as intracellular signaling molecules responsible for the pathophysiological effects in the cartilage [9,10,11], contributing to its erosion. With this function in view, targeting dysfunctional macrophages may be an interesting approach to improve the efficacy of RSV as anti-inflammatory and antioxidant agent by administering the drug to the inflammation site. In fact, current treatments involve a combination of drugs at different disease stages with the aim of controlling pain and fatigue, and blocking the inflammatory processes. Current therapies include non-steroidal anti-inflammatory drugs, corticosteroids, disease-modifying antirheumatic drugs, and biological drugs. However, curing inflammatory diseases affecting the joints is still out of our reach.

Several techniques are routinely used to produce polymeric microcapsules from polyesters, such as poly(ε-caprolactone) [12,13,14]. However, ultrasonic atomization as a method of obtaining microcapsules has not been so widely explored. As a consequence of its simplicity and versatility, coaxial ultrasonic atomizers have been shown to produce microcapsules in mild conditions, rapidly leading to a uniform size distribution, a spherical population with desirable properties of drug release, and high loading efficiencies, depending on procedure configuration. Some of the experimental parameters that can be set to obtain a controlled size distribution are the flow rates of the inner and outer channel, the power input provided by an ultrasonic generator, or the polymer concentration [15,16,17,18,19,20,21,22]. Using this technique, microdrops are generated in air as a soft spray by the ultrasound vibrational energy and the polymer coating around the aqueous inner core is solidified in a collection bath, where the organic solvent is removed by evaporation. One of the advantages of ultrasonic atomization over other conventional approaches is the fabrication of core-shell microcapsules in a few minutes. Likewise, the method offers the possibility of generating particles in a continuous manner, thereby allowing for a scale-up production. In the present work, we explore the influence of the encapsulation of RSV by ultrasonic atomization in a biocompatible polymer (PCL) on the stability and antioxidant properties of RSV. Following an optimization process, RSV-loaded microcapsules were prepared by ultrasonic atomization and characterized in terms of RSV encapsulation efficiency, particle size, morphology, RSV stability, antioxidant properties, and release. Finally, cell studies in activated mouse macrophage cultures were performed to examine the preservation of the biological activity of encapsulated RSV as an anti-inflammatory agent, with a potential use in joint disease treatments.

## 2. Materials and Methods

Poly(ε-caprolactone) (*M*_n_ = 14,000 g mol^−1^), *trans*-RSV, and PVA (poly(vinyl alcohol), *M*_w_ = 30,000–70,000 Da) were supplied by Sigma-Aldrich Química (Madrid, Spain). Dichloromethane and ethanol were purchased from Fluka (Sigma-Aldrich Química). All other reagents were of reagent grade.

### 2.1. Preparation of Microcapsules

Microcapsules were fabricated by using a coaxial ultrasonic atomizer nozzle (Sono-Tek Corp., Milton, NY, USA) equipped with a power supply (frequency: 60 KHz) and variable power. Briefly, the corresponding amount of PVA (polyvinyl alcohol) was dissolved in water (3.5%) and loaded into a syringe and infused at a constant flow rate of 3 mL/min through both channels, using a peristaltic pump (Masterflex 77390-00, Masterflex SE, Gelsenkirchen, Germany) for the inner channel and a programmable syringe pump (NE-1000; New Era Pump systems Inc., Farmingdale, NY, USA) for the outer channel. A fine and a stable spray was obtained and collected over a dichloromethane stirring solution containing RSV (3.3% *w*/*w*) and PCL (Table 1). Microcapsules were formed in suspension by the gradual precipitation of PCL and the solvent was evaporated using a rotavapor. Afterwards, microcapsules were washed and isolated (6000 rpm for 8 min; centrifuge EBA 21, Andreas Hettich GmbH & Co., Tuttlingen, Germany). Finally, particles were dried under vacuum for 24 h.

All experiments, except those for evaluation photodegradation of RSV within the microcapsules, were carried out under subdued light to prevent degradation of RSV. 

### 2.2. Experimental Design

To assess the effect of the polymer concentration and the power on the formation, morphology, loading, and release properties of the particles, an experimental design was used. RSV-loaded microcapsules were prepared in accordance with a central composite rotational design. To optimize the formulation and the evaluation of the effect of PCL concentration and power on the particle size and delivery rate, these two independent variables, at different levels in the experimental design, were used to systematically prepare the PCL formulations encapsulating the RSV (Table 1). The limits for PCL concentration were established between 0.08% and 2.91% and the power ranged between 3.58–6.41 W. The experimental design was built with Statgraphics Centurion XVI, v. 16.1.15 (StatPoint Technologies, Inc., Warrenton, VA, USA, 2010). PVA concentration and drug proportion were fixed at 3.5% and 3.3%, respectively. Limit values for the experimental design are displayed in Table 1.

The quantification of the effect of these variables on some microcapsule features were performed by the polynomial regression model and response-surface plotting, using the same software mentioned above.

### 2.3. Characterization of Microcapsules

#### 2.3.1. Determination of Drug Encapsulation Content

A total of 20 mg of microcapsules was dissolved in 10 mL of dichloromethane and stirred vigorously for 1 h. Then, the samples were filtered and diluted with ethanol. The loading efficiency of RSV in the microcapsules was then determined spectrophotometrically and fluorometrically by measuring at the maximum wavelength of 307 nm, and λ_exc_: 334 nm and λ_em_: 379 nm, respectively. The amount of encapsulated drug after the extraction procedure from the microcapsules was quantified as the percentage of encapsulated drug, with respect to the theoretical loaded RSV.

#### 2.3.2. Particle Size Analysis

Microcapsules were analyzed for their size distribution using laser diffraction in a particle size analyzer (Mastersizer Micro, Malvern Instruments, Malvern, UK) using a Fraunhofer diffraction model. The particles were analyzed after their final drying. The dried powder samples were resuspended in water and sonicated for 1 min before each measurement. The particle size distribution is presented in the volume-weighted mode and characterized by their 10%, 50%, and 90% undersize diameters (*d*(*v*, 0.1); *d*(*v*, 0.5); *d*(*v*, 0.9)). The 50% undersize diameter was used to express the mean diameter and *d*(*v*, 0.1) and *d*(*v*, 0.9) were used to calculate the span of the size distribution according to Equation (1):

(1) $$span=\frac{d(v,0.9)-d(v,0.1)}{d(v,0.5)}.$$

#### 2.3.3. SEM

The morphology of the microcapsules was observed using scanning electron microscopy (SEM; Jeol JSM-6360LV, JEOL Ltd., Akishima, Japan). Samples were mounted on double-sided tape, on aluminum stubs and sputter-coated with gold/palladium, and micrographs were taken at the appropriate magnification at the accelerating voltage of 15 KV. To observe the cross-section of microcapsules, particles were dispersed into ethyl-2-cyanoacrylate pellets and then sectioned using a razor blade.

#### 2.3.4. DSC

The thermal properties of the optimized formulation of RSV-loaded microcapsules, empty microcapsules and RSV were determined by Differential Scanning Calorimetry (DSC). The measurements were carried out in triplicate, using a DSC (Mettler-Toledo International, Columbus, OH, USA). All the experiments were performed from 25 to 300 °C at a scanning rate of 10 °C/min. The equipment was calibrated for baseline using indium as the standard. The experiments were performed using non-hermetic aluminum pans, in which 5–10 mg representative samples were weighed and covered with a lid. 

#### 2.3.5. Photodegradation

To test RSV stability in free form and its potential protection in encapsulated form, *trans*-RSV was placed in quartz cuvette and UV-irradiated directly using a lamp model operating at 254 nm (Vilver Lourmat, VL-204G, Marne-la-Vallée, France). A concentrated stock solution of RSV was prepared to obtain the calibration curves. Diluted standard solutions, over the range of 0.5–7 μg/mL, were analyzed by UV-visible spectrophotometry (Evolution 60S, Thermo Fisher Scientific, Waltham, MA, USA). Degradation of RSV dissolution was assessed by the corresponding calibration curve (*y* = 0.0024 + 0.1311*x*; *r*^2^ = 0.9999). The molar absorptivity of the *trans*-RSV solution, absorbing at 307 nm, was 29924 M^−1^ cm^−1^ (*r*^2^ = 0.9999). Additionally, the experiments were carried out using pure RSV in a powder form and an equal quantity of RSV was encapsulated in PCL as the dried powder irradiated with a UV lamp (λ = 254 nm) at a 20 cm distance from samples, for 3.2 h. After that period, samples were dissolved in ethanol and centrifuged. RSV was quantified using UV spectrophotometry (λ = 307 nm). For comparison, the stability study of RSV samples was performed in water and ethanol.

#### 2.3.6. ABTS Radical Scavenging Activity

The antioxidant activity of free RSV and RSV-loaded in microcapsules was evaluated using the ABTS assay, according to the method described by Re et al. [23]. An ABTS radical decolorization assay was used to determine the antioxidant activity of RSV. In this method, RSV, as antioxidant molecule, was added to a pre-formed ABTS radical (ABTS^•+^) solution and, after 40 min, the remaining ABTS^•+^ was quantified spectrophotometrically at 730 nm. Briefly, ABTS (2,2′-Azino-bis(3-ethylbenzothiazoline-6-sulfonic acid) diammonium salt) was dissolved in water (7 mM) and later the ABTS radical cation (ABTS^•+^) was generated by mixing with 2.45 mM potassium persulfate. The mixture was left for reacting for 16 h before use and was protected from light. Different concentrations of RSV and RSV samples extracted from the microcapsules were reacted to ABTS^•+^, which was diluted previously with ethanol to an absorbance of 0.8 ± 0.02, at 730 nm. Solutions and suspensions of RSV and microcapsules were added to the radical cation solution in such a way that, after the mixture of 3.7 mL of ABTS plus 0.3 mL of samples, a 20–80% reduction of blank absorbance was obtained. The time of incubation for the complete reaction was fixed at 2 and 40 min, for Trolox and RSV, respectively. The percentage of inhibition of absorbance, at 730 nm, was calculated as a function of the concentration of antioxidants and Trolox (Sigma Aldrich) (6-hydroxy-2,5,7,8-tet-ramethychroman-2-carboxylic acid), used as an antioxidant standard. Thus, the antioxidant activity of RSV is expressed in terms of Trolox Equivalent Antioxidant Capacity (TEAC). For the assay of loaded RSV, 15 mg of microcapsules were weighted, dispersed in 10 mL of ethanol, and maintained under stirring (750 rpm) for 1 h. Samples were centrifuged at 6000 rpm for 5 min. An ABTS assay was performed as described above, using a range of concentrations between 0.035 and 0.125 mM from RSV extracted from microcapsules. Empty microcapsules were used as negative control. All samples were diluted in ethanol.

#### 2.3.7. In Vitro Release Profile

For release studies, 5 mg of RSV-loaded microcapsules were suspended in 25 mL of buffer solution and placed in borosilicate vials, then incubated at 37 °C and stirred in an orbital mixer (Unimax 1010/Inkubator 1000; Heidolph Instruments, Schwabach, Germany, 200 rpm). Release tests were carried out in various buffers, as follows: PBS (50 mM, pH 7.4), PBS (50 mM, pH 6.4), and acetate buffer (100 mM, pH 5) for 485 h. The experiments were carried out under dark conditions and the samples were always protected from light exposure. 

Samples were withdrawn at predetermined time intervals and replaced with an equal volume of fresh medium. Sink conditions were kept during the whole experiment time. As controls, an RSV standard solution of 10 µg/mL and blank particle suspension were maintained in the same conditions to determine whether degradation from particles or the own degradation of RSV could interfere in the analytical determination of the RSV. Finally, samples were centrifuged and assayed by fluorimetry. The concentration of RSV in the release samples was quantified using wavelengths of 334 and 379 nm (excitation and emission, respectively). The equation of the obtained calibration curve was *y* = 0.0006 + 0.1353*x* (*r*^2^ = 0.9999). In addition, to determine RSV stability in the release medium, the degradation of RSV was investigated in the three different buffer mediums (pH 5, 6.4, and 7.4) under dark conditions. For determination of the stability of RSV in aqueous samples over the time release, the UV spectra of RSV were acquired in the range of 200–400 nm. 

### 2.4. Cell Culture and Treatments

Mouse macrophage cell line RAW 264.7 was obtained from ECACC (RAW 264.7; ECACC 91062702, Salisbury, UK). Macrophages were cultivated in Dulbecco’s Modified Eagle’s Medium (DMEM; Sigma Aldrich), supplemented with 10% inactivated Fetal Bovine Serum (FBS) and 1% antibiotic/antimycotic mixture, consisting of 10,000 units/mL penicillin and 10,000 units/mL streptomycin in 0.85% saline (Chemicon International, Thermo Fisher Scientific). Macrophages were cultured in a humidified 5% CO_2_ atmosphere at 37 °C.

### 2.5. Cell Proliferation Assay 

The microcapsule cytocompatibility was evaluated by two standard methods, the WST-1 assay and a neutral red uptake test. For WST-1 assay, cells were seeded in 96-well plates at a density of 15,000 cells/well in DMEM supplemented with 10% FBS and left overnight for attachment. Cells were co-incubated with 4 different RSV concentrations, with blank and RSV-loaded microcapsules (10 and 15 µg per well). Triplicate cultures were maintained for 24 h for each treatment. Cells proliferation was determined using the WST-1 reagent (Roche Applied Bioscience, Madrid, Spain), according to the manufacturer’s protocol.

The neutral red uptake test was performed as follows: A neutral red stock solution of 40 µg/mL was added to the cell culture (10,000 cells/well) in 96 well plates, overnight. A de-stain solution for neutral red (50% ethanol: 49% deionized water: 1% glacial acetic acid) was used. For quantifying the extracted neutral red, a calibration curve was included in each experiment.

### 2.6. Determination of NO Production

The level of nitric oxide (NO) was measured indirectly through the accumulation of nitrite in the cell culture supernatant produced by the macrophages. NO was measured by using the Griess method upon co-incubation without and with loaded RSV. Supernatant from the cell cultures was incubated with an equal volume of the Griess reagent mixture (1% sulfanilamide, 0.1% *N*-(1-naphtyl)-ethylenediamine dihydrochloride, 2.5% H_3_PO_4_) at room temperature for 10 min. The absorbance was measured in a microplate reader (Multiskan microplate spectrophotometer, Thermo Scientific). A stock solution of lipopolysaccharide (LPS; 1 mg/mL) from *E. coli* serotype 0111.B4 (Sigma Aldrich) was prepared in PBS (pH 7.4) and stored at −20 °C until use. RAW macrophages were left untreated (negative control), incubated with LPS (0.5 μg/mL; positive control), or pre-stimulated with LPS before the co-incubation with RSV microcapsules. For comparison, the RSV concentrations investigated in the cell cultures were 1, 10, and 50 µM, both in free form and encapsulated in microcapsules. 

### 2.7. Determination of Intracellular Reactive Oxygen Species

The detection of reactive oxygen species in the RAW macrophages were determined by a fluorometric microplate assay based on the oxidation of 2′,7′-dichlorofluorescein diacetate (DCFH-DA). For testing the reactive oxygen species in macrophages upon incubation with microcapsules, cells were harvested and plated in phenol red-free medium in 96 well black flat bottom plates at a density of 25,000 cell/well. The cells were then incubated with 20 µM PBS of DCF-DA for 45 min and then incubated afterwards, with 10 µg of microcapsules per well in a humidified atmosphere (5% CO_2_, 37 °C). Respiratory burst was assessed by measuring the cumulative production of 2′,7′-dichlorofluorescein with a fluorescence microplate reader (λ_exc_ and λ_em_ of 495 and 529 nm, respectively; Fluostar Optima, BMG labtech, Ortenberg, Germany)). Free RSV, blank microcapsules, and RSV-loaded microcapsule-induced ROS production was expressed as the percentage of fluorescence relative to a positive control of cells stimulated with zymosan A particles (0.5 mg/mL; Sigma-Aldrich) and after background subtraction. Untreated cells were used as a negative control.

### 2.8. Phagocytosis Assay

Studies were performed in RAW 264.7 mice macrophages. Macrophages cultured in 24 well plates (5 × 10^5^ cells/well) were co-incubated with 50 µg/mL of particles (*n* = 6). For the assessment of phagocytosis, microspheres dispersed in a low concentration serum were added and incubated at 37 °C in a 5% CO_2_-humidified atmosphere for 4 h. The medium was exchanged to remove non-phagocytosed particles and fixed with methanol for Giemsa staining. Cells were observed for phagocytosis by light microscopy (Olympus IX71, Olympus Corp. Tokyo, Japan). Ten randomly acquired images were taken from ten samples at 20- and 40-fold magnification, using a CCD camera (DP 71, Olympus). The number of phagocytosed microcapsules was assessed microscopically by counting the number of macrophages (200 cells) with at least one microcapsule being uptaken (phagocytosis index).

### 2.9. Lipid Peroxidation Thiobarbituric Acid Assay

The antioxidant activity of the RSV formulations in cell cultures was evaluated using the Thiobarbituric Acid Reactive Substances (TBARS), according to the method of Vyncke (1970). The TBARS was determined as follows: The cells (2 × 10^6^) were washed twice with a lysis buffer (Tris-HCl, KCl 150 mM) and lysed by using ultrasonication on ice. Cell lysate samples were mixed with TCA solution (trichloroacetic acid; 10%) and thiobarbituric acid solution (0.6%). Subsequently, the mixture was heated at 95 °C for 60 min. After centrifugation of precipitated proteins, TBARS content was determined by measuring the absorbance at 532 nm. A calibration curve, using malonaldehyde as a standard, was performed. The appropriate volumes of standards were added to the TCA solution under the same conditions used for the samples, as described above. Macrophages were exposed to oxidative stress (positive controls) by incubating 100 µM and 5 mM of *tert*-BHT (tertiary butylhydroperoxide) and H_2_O_2_ as positive controls. Protein concentration of the cell lysates was determined using a plate assay, according to BCA assay. To test the efficacy of RSV and RSV-loaded microcapsules, macrophages were pre-treated with oxidative stress inducers (H_2_O_2_ and *tert*-butyl hydroperoxide) and then co-incubated with the formulations. The RSV concentration tested in the cell cultures was always 10 µM, in both free form and as encapsulated. Results were expressed in concentration of malonaldehyde, nM/mg protein).

### 2.10. Statistical Analysis of the Data

All experiments were performed in triplicate and the final results have been reported as the mean ± standard error of the mean. Statistical analysis of the data was performed using the Statgraphics Centurion XVI, v. 16.1.15 (StatPoint Technologies). Statistical comparisons were made using one-way analysis of variance (ANOVA), followed by a Tukey–Kramer test for multiple comparisons, and differences were considered significant at *p* ≤ 0.05.

## 3. Results and Discussion

### 3.1. Experimental Design

The selected ranges for the nozzle power and the polymer concentration given by the experimental design were 3.58–6.414 Watts (W) and 0.08–2.91% of PCL. All formulations were obtained under these conditions. However, higher concentrations of the polymer or higher or lower power caused nozzle clogging or no particles were obtained, which resulted in complete PCL insolubilization and agglomeration in the aqueous medium.

To investigate the influence of each factor (power and % PCL) on particle size and drug release, the causal factor and the independent variables were related by multiple regression (Equation (2)) and statistical analysis. Furthermore, the response surface methodology was used to search for the optimal RSV formulation. The relationship among variables was as follows:

(2) Mean size(0.5)=−7.59378+2.64044×W+10.6761×%PCL

**Figure 2:**
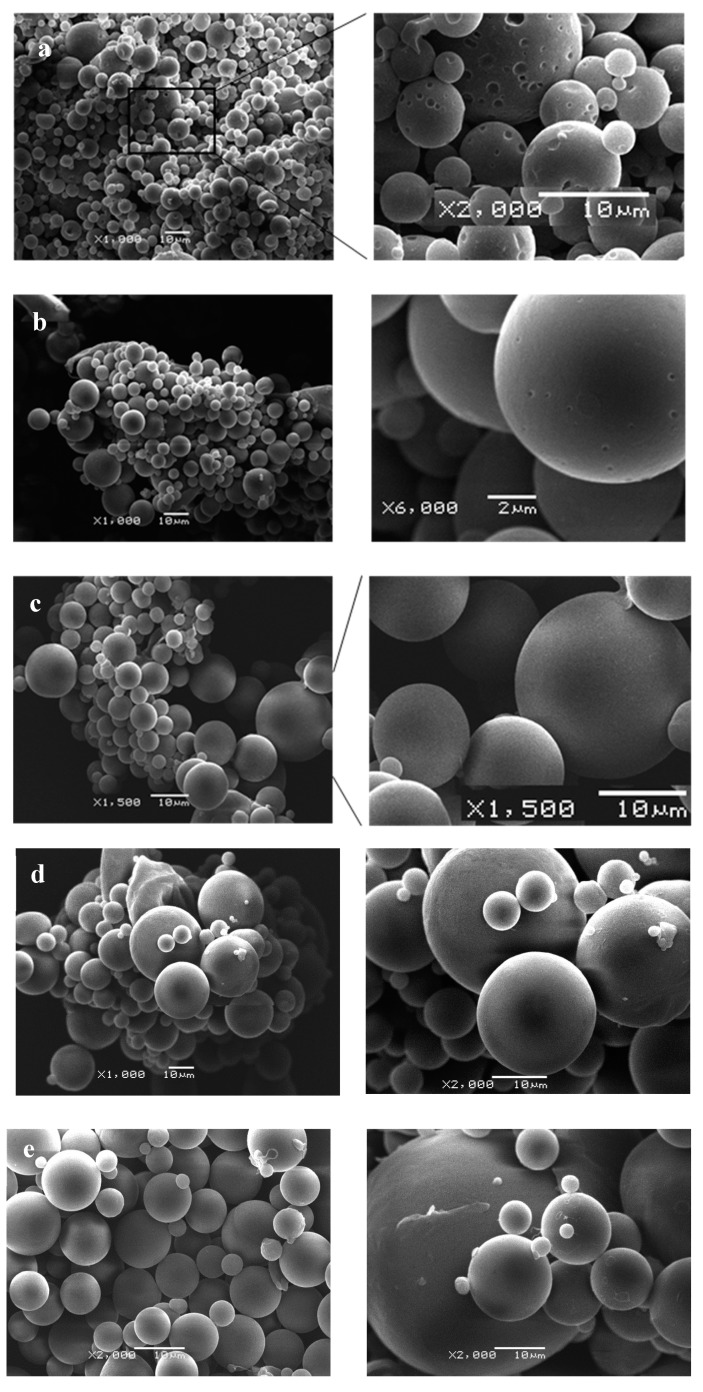
Representative Scanning electron microscopy (SEM) micrographs of RSV-loaded microcapsules: (**a**) F1 (0.08% PCL; 5 W); (**b**) F3 (0.5% PCL; 6 W); (**c**) F5 (1.5% PCL; 5 W); (**d**) F7 (2.5% PCL; 4 W); (**e**) F9 (2.91% PCL; 5 W).

The statistical analysis (ANOVA) deduced, from the calculated response with the multiple regression model (*p*-value = 0.0128) and the values for particle size obtained experimentally, that there was a statistically significant relationship, with a confidence level of 99.97 between the independent variables and the response determined (particle size), although only 58% of variability on particle size could be explained (*r*^2^ = 0.581) by changes in the nozzle power (W) and percentage of PCL. In addition, according to this polynomial equation, % PCL played a critical role on particle size, rather than the power used to create the ultrasonic spray (see Figure 2). In light of these results (size and encapsulation efficiency), the so-called F5 formulation was selected for further studies.

### 3.2. Preparation of Microcapsules

Double emulsion-solvent evaporation techniques are the most used techniques to obtain drug-loaded microcapsules. These methods are difficult to scale-up and include many differences in formulation parameters, which may lead to a lack of reproducibility between laboratories. In addition, depending on the molecule, these techniques have a few critical disadvantages when the drug is especially unstable (proteins, photostability, etc.), including the low encapsulation efficiencies that are typically obtained and the impossibility of obtaining large batches. In this study, in an attempt to reduce some of these drawbacks, RSV was encapsulated in PCL as a wall-forming material, around an aqueous nucleus obtained by ultrasonic atomization using a dual feed coaxial nozzle. An ultrasonic atomizer generates a stable, soft, and low velocity and fine spray by creating capillary waves using high frequency sound vibrations. The main feature of this technology relies on the control of the mist spray produced by the nozzle (without temperature and without pressure) and subsequently, the final particle size. In addition, this technology provides a uniform, spherical, and narrow microparticle size by controlling the liquid delivery system (flow rate) in combination with the nozzle and the input power used during the encapsulation process, when operating at fixed frequencies (60 KHz). Although this process has been carried out at an initial lab-scale, the well-controllable preparation of microcapsules makes this technology feasible to produce large quantities of particles in a continuous manner. The interest in using an antioxidant as RSV relies precisely in the need to fabricate particles that ensure its stability against photodegradation, in a large enough quantity and at a quality suitable for a biomedical application, for local administration in inflammatory sites.

The encapsulation efficiency of the microcapsule formulations varied between 39.66–96.0% (Table 1), depending of the composition. Bearing in mind the lipophilic nature of RSV, the high encapsulating efficiency is not surprising. 

In this work, PCL microcapsules were fabricated using coaxial ultrasonic atomization to generate single-walled microcapsules containing RSV. The fabrication of RSV-loaded microcapsules by ultrasonic atomization rendered a high process yield (Table 1). Depending on the formulation, the yield percentage varied from 80.22% to 92.07%. This fact confirmed the good reproducibility and reliability of this technology. Furthermore, apparently the high process yield is not dependent on the polymer concentration or the input power used during the process, at least in small batches.

### 3.3. Characterization of Microcapsules

#### 3.3.1. Encapsulation Efficiency, Particle Size and Morphology (SEM)

Regarding particle size, the results showed that the concentration of the polymer greatly affected the particle size (Figure 2). Concisely, the particle size for the optimized formulation was *d*(*v*, 0.1): 1.96 ± 0.16 μm; *d*(*v*, 0.5): 13.82 ± 0.3 μm, and *d*(*v*, 0.9): 29.76 ± 1.79 μm. Thus, the sphericity, as well as the mean size (also taking into account the dispersion of mean size), obtained for the selected formulation for the ongoing cellular studies was considered as appropriate for the intra-articular injection, as Pradal et al. have demonstrated in in vivo studies where the biodistribution of particles of various sizes were assessed after intra-articular injection [24]. These authors have demonstrated that the administration of relatively large particles (10 µm) was retained in the inflammation site, thus prolonging the release of a model drug and skipping the leakage from the joint.

**Figure 3:**
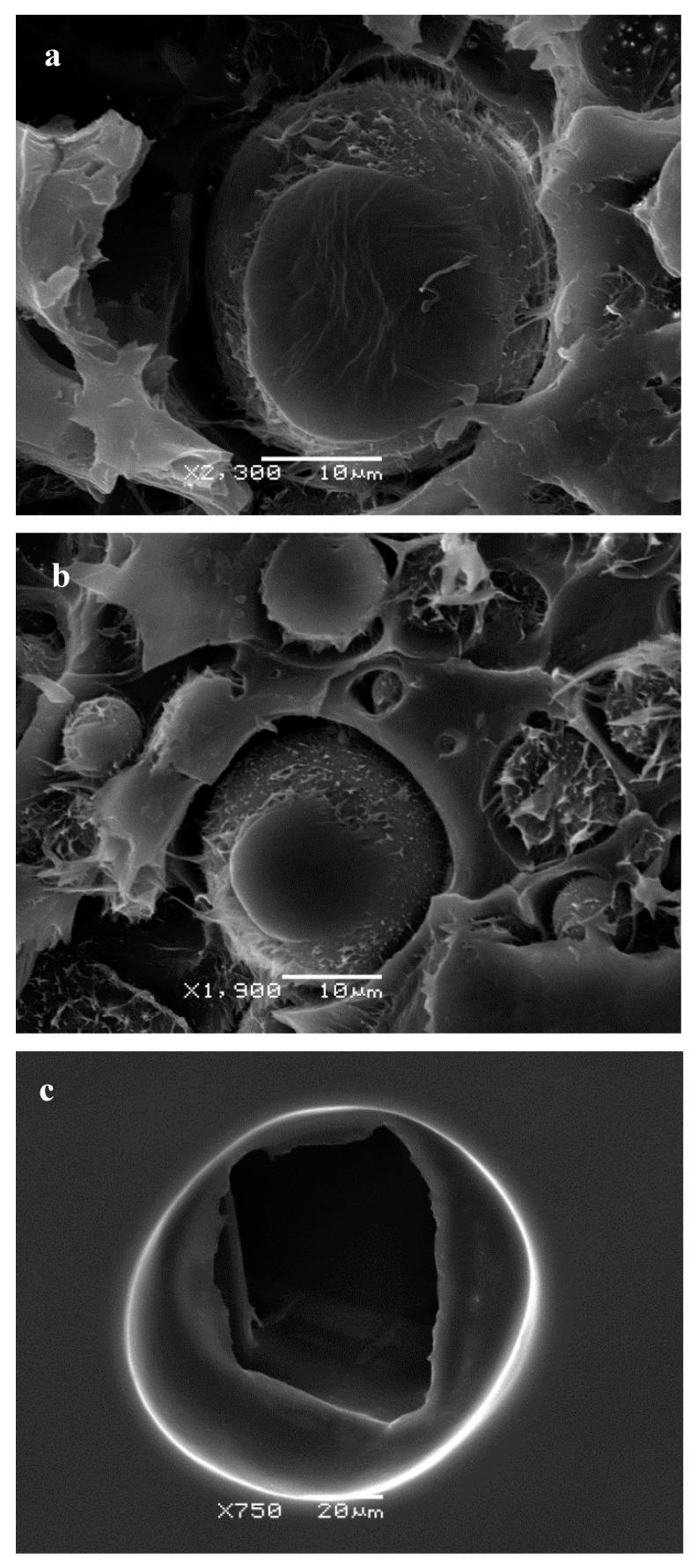
Representative SEM micrographs of cross-section RSV-loaded microcapsules prepared with different concentrations of poly-ε-caprolactone: (**a**) 2.91% PCL; (**b**) 1.5% PCL; (**c**) 0.08% PCL.

A typical light microscopy image of microcapsule suspension is shown in Figure 3. PCL microcapsules were spherical and slightly/fairly polydisperse and displayed diameter sizes in consonance with those obtained by light scattering measurements. No aggregation during observation was detected and they could be dispersed in water by hand shaking.

SEM observation of microcapsules revealed the surface morphology was smooth and spherical for all formulations, although a great effect of polymer concentration was observed with changes in surface morphology. Clearly, the lower the poly-ε-caprolactone concentrations, the higher the irregularity on the surfaces (showing porous outer surface). As shown in the micrographs displayed in Figure 3, the microporous feature of the microcapsule’s surface is increased as the content decreases, which can be attributed to the effect of the rapid evaporation of CH_2_Cl_2_ from the low-concentration polymer of these microcapsules. The fractured microcapsules clearly showed a dense polymeric wall surrounding an inner empty space and, apparently, no differences in the thickness of the polymeric walls were found when compared with the PCL concentration (Figure 3). Additionally, the inner structure of the microcapsules revealed a hollow structure with a cavity without internal porosity (Figure 3). No drugs crystals were observed on the surface of the microcapsules. 

#### 3.3.2. Characterization of the Physical State of RSV in Microcapsules (DSC)

**Figure 4:**
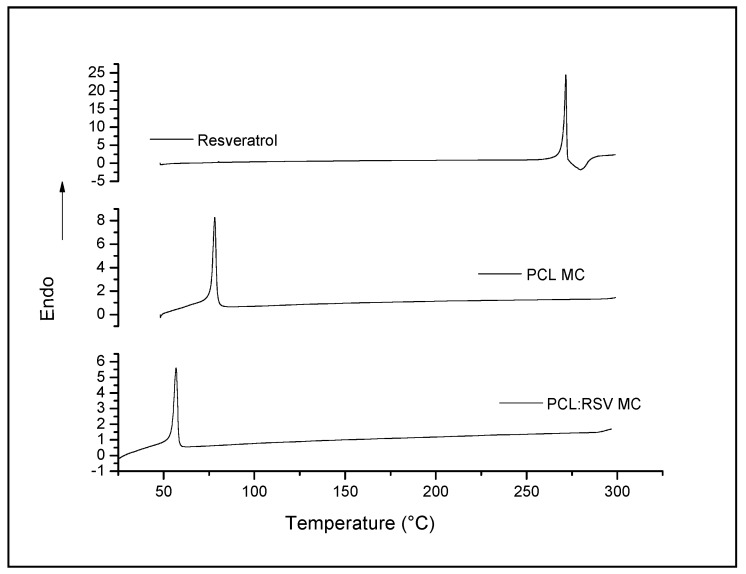
DSC curves obtained from pure RSV, empty micro capsules, and PCL microcapsules containing RSV.

The calorimetric characterization of RSV and polymer:drug microcapsules, prepared by ultrasonication, was investigated in order to identify the physical state of RSV within the microcapsules (Figure 4). The RSV DSC curve showed a sharp and large peak (exotherm) corresponding to a melting point of 267 °C (Δ*H* = 200 J/g). However, the typical melting peak of the drug was not present on the DSC curve of the microcapsules containing RSV, which indicated that the drug was dispersed in the polymeric coating wall of the microcapsules as an amorphous form. In addition, microcapsules exhibited one peak at 58 °C, attributed to the melting point of PCL, confirming previous data for the polymer [25]. The results concerning empty microcapsules confirmed that there is no great change in the melting point of the polymer.

Poly-(ε-caprolactone) is a biodegradable and semi-crystalline polymer with a low melting point (*T_m_)* of 68.3 °C and a glass transition temperature (*T_g_*) of −63 °C. Owing to the hydrophobic character and semi-crystalline nature of PCL, it is known that the degradation is very slow in an aqueous medium. Therefore, this fact suggested that RSV release was due to a diffusion mechanism, rather than the degradation of the polymer. These results were in agreement with the release rate of RSV at pH 5 and the best fitting to a mechanism of release (see below).

#### 3.3.3. Photodegradation and Stability

The low stability, poor solubility, and bioavailability of *trans*-RSV have limited its biomedical applications. This fact brought forth the need to encapsulate it in order to protect the molecule against photodegradation, as well as the enhancement of RSV concentration in the site of action via sustained release. 

The limited stability of RSV upon the exposure of UV to *trans*-RSV is widely known. In fact, in this condition, the first step is the isomerization of the molecule to *cis*-form, followed by a complex generation of transformation species produced by the oxidation, hydroxylation, or even the fragmentation of the subsequent molecules [26,27].

**Figure 5:**
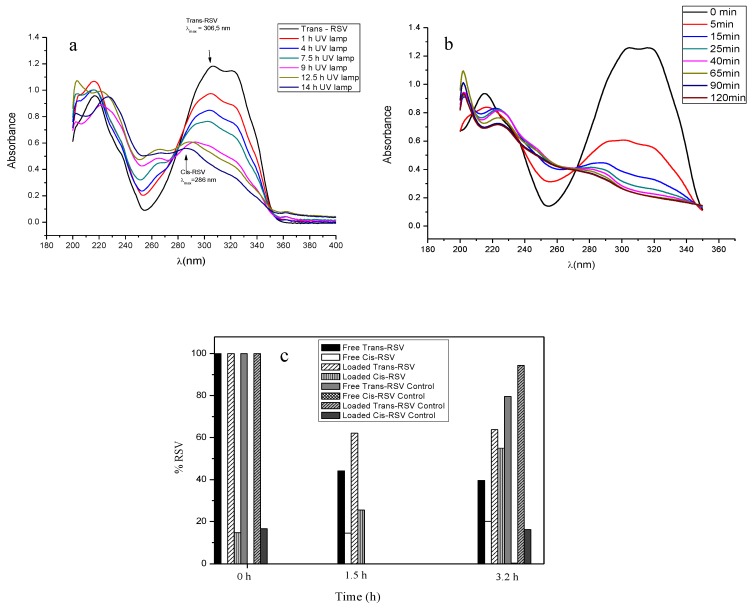
UV-visible spectra of isomerization and degradation of RSV under UV light exposure in (**a**) ethanol and (**b**) PBS (50 mM), pH 7.4. (**c**) Photodegradation study in solid: Percentage of the decay of *trans*-RSV, free and encapsulated, in microcapsules after UV light exposure (254 nm).

In this study, the protective effect of the encapsulation of RSV against photo-degradation, compared to free RSV, has been examined in both a solution and a solid form (Figure 5). Concerning experiments in a solid form, a comparison was made between free RSV (powder) exposed to UV irradiation and protected from light and the same amount of RSV (loaded in microcapsules), exactly in the same experimental conditions (dark and with UV light exposure). As it is shown, the encapsulation of RSV in PCL microcapsules enhanced its photostability under UV exposure. The exact mechanism underlining the degradation of RSV in solid form (PCL microcapsules) at longer times (3.2 h) is not completely understood, since the degradation of molecules near the surface would likely be faster than those located deeper within the PCL coating. The *cis*-form represents a main intermediate, whereas has been demonstrated that, at similar experimental conditions to transform *trans* into *cis*-RSV, a third compound was observed when the concentration of *trans*-RSV was below 100 mg/L [28], thus indicating the dependence of the reaction kinetics of the working initial concentration of RSV. It must be noted that no *cis*-RSV isomer was found under dark conditions. By contrast, exposure to UV light led to an increase in the *cis*-RSV proportion, although in this case the exact amount of *cis*-RSV was not completely defined, since another transformation product (not identified) was detected with the presence of overlapping in the spectra. Therefore, the percentage of *cis*-form displayed is not completely determined by UV visible spectrophotometry. 

Here, we also study the physicochemical properties of *trans*-RSV in solution, in different media, to better comprehend how the encapsulation process and the release could affect the isomerization of the molecule from *trans*-RSV to *cis*-RSV (more stable but less biologically active than *trans*-RSV) [27]. The studies were performed under UV light, since the isomerization is known to be affected by the wavelength and the irradiation time [6] compared with the same RSV concentrated standards under the same conditions of temperature and pH but protected from light.

**Figure 6:**
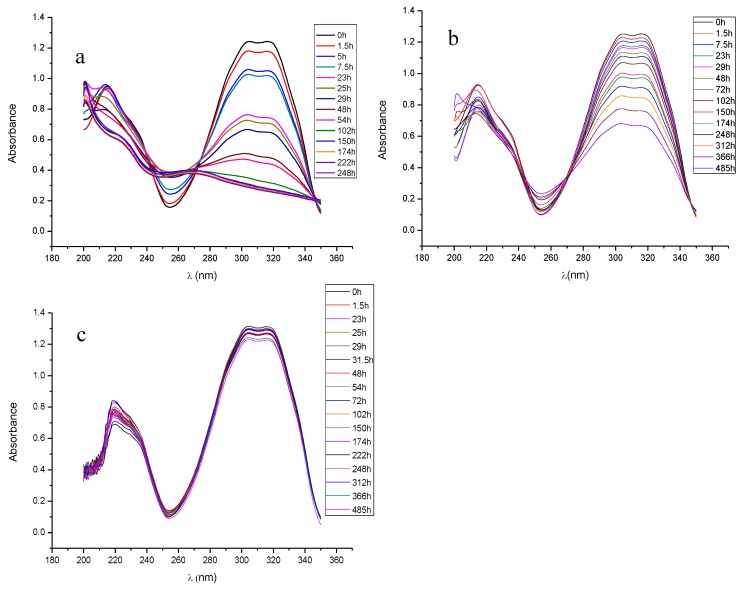
Degradation of *trans*-RSV in solution at (**a**) pH 7.4, (**b**) pH 6.4, and (**c**) pH 5 buffer, under dark conditions.

The isomerization and degradation of *trans*-RSV to the *cis*-form of a UV-irradiated solution of *trans*-RSV in ethanol and PBS, respectively, is displayed in Figure 5a,b. Secondary absorbance maxima were observed for both isomer forms. Concisely, in ethanol, the percentage of each isomeric form was 50% after 4 h of exposure. Regarding the stability of RSV in aqueous solution under dark conditions, the *trans*-RSV degradation is clearly affected by the pH level of the solution (Figure 6). For comparison, results indicated that *trans*-RSV has a rapid isomerization in PBS (pH 7.4) when the samples were exposed to UV light, in contrast to the same concentration of samples that were protected from light (Figure 6a). Furthermore, the lineal fit of RSV isomerization from *trans*- to *cis*- form followed first order kinetics (*y* = −0.0856*X* + 1.013; *r*^2^ = 0.9975), as confirmed by the comparison between the RSV concentration in function of the UV exposure time. As can be observed, upon UV irradiation, a degradation product of RSV appeared at 260 nm (Figure 5a), indicating a photodecomposition process of the RSV molecule. In consonance with other authors, RSV was found to be stable for much longer times at lower pH buffers (6.4 and 5) and under dark conditions [6,29] (Figure 6b,c). In fact, the stability of RSV solutions at pH 5 was kept for at least 312 h. A very complete study of the kinetics of RSV degradation in aqueous solutions, as a function of temperature and pH level, has been already published [30].

In summary, as expected, the proven physical and structural stability of PCL microcapsules not only provided prolonged chemical stability of RSV but, additionally, the biological properties attributed to *trans*-RSV were preserved.

#### 3.3.4. ABTS Radical Scavenging Activity of RSV

In this work, polymeric microcapsules were used to provide protection against RSV degradation (chemical stability) and, as a consequence, the loss of its antioxidant ability. As ABTS assay was used to determine the ability of RSV for scavenging the excess production of reactive oxygen species that lead to inflammation in biological situations, such as inflammatory diseases. To determine the antioxidant activity of RSV, a linear relationship was obtained between the extent of the reduction of the radical cation ABTS^•+^ as a function of concentration of RSV (free drug and loaded within microcapsules) and Trolox. Thus, the linear relationship between free RSV and the percentage of reduced ABTS^•+^ was confirmed by the following linear regression: *y* = 0.2321 + 4.778[RSV]; *r*^2^ = 0.9972). To calculate the TEAC (Trolox Equivalent Antioxidant Activity), a calibration curve of Trolox concentration versus percentage of degraded ABTS^•+^ was obtained in a range of stock drug concentrations, from 0.07–0.45 mM (*y* = 0.0387 + 1.7741*x*; *r*^2^ = 0.9987). Additionally, a calibration curve for using RSV alone was also obtained with RSV standards in a range of concentrations, from 0.04–0.9 mM (*y* = 0.2321 + 4.4778*x*; *r*^2^ = 0.9972). Furthermore, in order to determine the antioxidant activity of loaded RSV, a calibration curve was obtained using the drug extracted from microcapsules and ABTS. The concentration range of solutions of RSV extracted from microcapsules varied between 0.035–0.125 mM. The curve obtained to relate the effect of RSV concentration on ABTS^•+^ reduction was as follows: *y* = 0.2783 + 3.5426[RSV]; *r*^2^ = 0.9922). 

In order to make the results of antioxidant activity on RSV comparable, two concentration-response curves were obtained by calculating the number of mmoles of RSV (free and encapsulated) equivalent to Trolox (mM) that produced the same percentage on ABTS^•+^ radical cation reduction. A relationship was obtained when different concentrations of RSV (free and encapsulated) and Trolox standards were assayed. This linear relationship gave a concentration of Trolox producing the same percentage reduction of ABTS radical as 1 mM of RSV. Thus, the estimation of free RSV concentration that gave the same percentage as the ABTS radical reduction was given as follows: [Trolox] = 0.109 + 2.5243[free RSV] (*r*^2^ = 0.9971), whereas the linear equation for estimating the antioxidant behaviour of loaded RSV was given by the following: [Trolox] = 0.135 + 2.0694[loaded RSV] (*r*^2^ = 0.9922). Therefore, the antioxidant activity obtained for RSV was 2.5243 and 2.07 (free and loaded RSV, respectively), expressed as TEAC values or Trolox equivalents. No statistical significance was found for the difference between both values of antioxidant activity. Furthermore, the linear relationship found between the concentration and antioxidant activity of RSV, compared to Trolox, is indicative of the good linearity of the method. Hence, the TEAC values achieved for the RSV and Trolox standards indicate the preservation of the antioxidant activity of loaded RSV at 82.14%, compared to free RSV. This finding evidenced the protection of RSV as a radical scavenger conferred by microencapsulation and demonstrates the potential efficacy of the antioxidant role of RSV-loaded microcapsules to mitigate the oxidation process present in inflammatory joint diseases. Nevertheless, the determination of ABTS reduction was also used with empty microcapsules and any interference could be ruled out, so the lower value obtained for loaded RSV could be due to the incomplete extraction of RSV or, most likely, to a certain percentage of isomerization or degradation that could take place during the extraction procedure. The in solution antioxidant activity of *cis*-RSV or other transformation products, with respect to the proportion of *trans*-RSV, remains to be fully elucidated.

#### 3.3.5. In Vitro Release Rate

**Figure 7:**
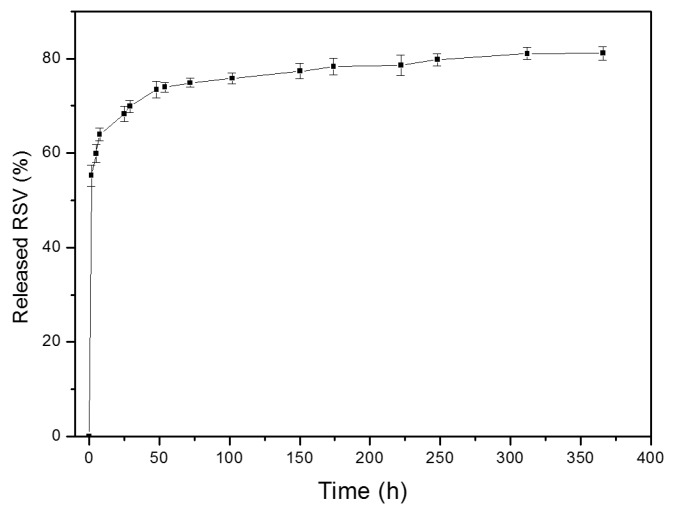
In vitro release profile of RSV at pH 5 and 37 °C under dark conditions.

The in vitro release of RSV was carried out at three different pH values (5, 6.4, and 7.4). However, here we present the results corresponding to release at pH 5 due to the lack of stability of RSV at pH 6.4 and 7.4, as stability and release studies at the other two pH have been demonstrated (data not shown). In fact, at pH 7.4, RSV was always degrading continuously while it was released from the microcapsules. The in vitro release at pH 5 is depicted in Figure 7. After an initial burst of over 55% release in the first 90 min, RSV was delivered in a sustained manner from microcapsules until the first signs of degradation were detected at 485 h. At that moment, more than 80% of the loaded amount of RSV had been released. These results support the fact that, at 24 h, approximately 68% of RSV was nearly released from microcapsules in cell culture experiments. However, the real exposure of RSV to macrophages is certainly difficult to assess, bearing in mind the complex behaviour of RSV at a physiological pH and in the presence of serum proteins during cell experiments.

Finally, to investigate the release of RSV of PCL microcapsules, the release data were fitted to a kinetic equation according to several mathematical models. According to this, the best fitting equation to the RSV profile release was Peppas’s equation ($Q=k\times t^{n}$), where *Q* is the percentage of drug released at each time (*t*), *k* is a constant which incorporates the structural characteristics of the release system, and *n* is the release exponent, indicative of the release mechanism. Thus, data were tested by one-way analysis of variance (statistically significant; *p* < 0.05) with a correlation coefficient of 0.9992. The equation obtained for the kinetic model was $Q=0.52254\times t^{0.09576}$, therefore indicating that the release of RSV took place predominantly by diffusion and it started when the formulation made contact with the release medium. After an initial release of the most accessible molecules of RSV to the medium, the low degradability of the polymer slowed down the release rate [31,32].

### 3.4. Cell Culture Assays

#### In Vitro Cell Viability 

**Figure 8:**
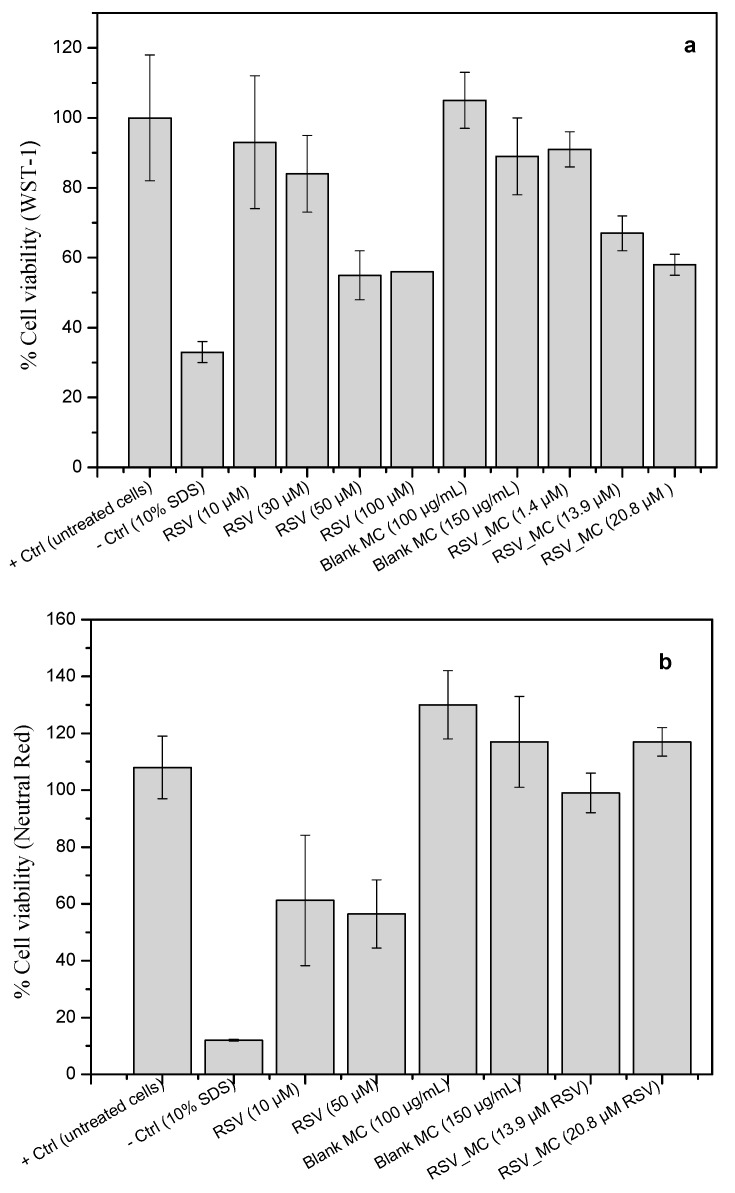
Percentage of macrophage viability upon incubation with free RSV, blank microcapsules, and RSV-loaded microcapsules at different concentrations. (**a**) WST-1 assay and (**b**) neutral red assay.

The WST-1 assay showed no statistically significant influence of 24 h exposure to blank microcapsules on the viability of RAW macrophages. Viabilities of 95–100% were recorded even at the highest concentration (Figure 8a). However, a direct effect with the concentration was observed on cell viability when cells were co-incubated with RSV and RSV-loaded microcapsules, therefore suggesting an antiproliferative effect of RSV at higher concentrations on mouse macrophages, compared to control cells, with a level of viability of 55%. These results are in agreement with inhibitory effects exhibited by RSV in a variety of cells types, as previously reported [33,34,35]. In contrast, other studies have shown no effect on viability on different macrophage preparations and using a similar range of RSV concentrations (10, 50, 100 µM) [36,37]. To get more insight in this issue, a neutral red assay was also performed, with similar results for RSV treatment (Figure 8b). A higher percentage of cell viability was observed when encapsulated RSV was co-incubated with macrophages (nearly 100%), which suggested that most of cells remained viable, as the uptake of the dye indicated. It is worthwhile to mention that different cell proliferation and viability assays have been used in each laboratory, which can justify the contradictory results concerning the cellular toxicity of RSV found in the literature. In summary, bearing in mind the amount of RSV encapsulated in microcapsules is corresponds to 7 µM (nominal content: 32 µg/mg microcapsules), certain protection was conferred by microcapsules against RSV. Overall, further research is required, taking into consideration that RSV, as with other polyphenols, is known to bind by complexation to some proteins, like BSA, always present in the cell culture medium [38]. Therefore, the real RSV concentration in the cell medium during the experiments was not completely elucidated.

### 3.5. Determination of NO Production

Nitric oxide (NO) is one of the most important chemical species that is considered a biomarker of oxidative stress, since it contributes to the development of inflammation [39]. RSV is known for its anti-inflammatory activity and, in fact, RSV has been reported to be involved in several cellular signaling events during inflammation stages, such as the nitric oxide synthase route [40,41]. Taking use of this fact, the approach of reducing the number of activated macrophages to inhibit the inflammatory signals of their specific products (nitric oxide, cytokines, and prostaglandins) that amplify the response, seems to be a rational strategy to retard the progression of chronic inflammation. 

**Figure 9:**
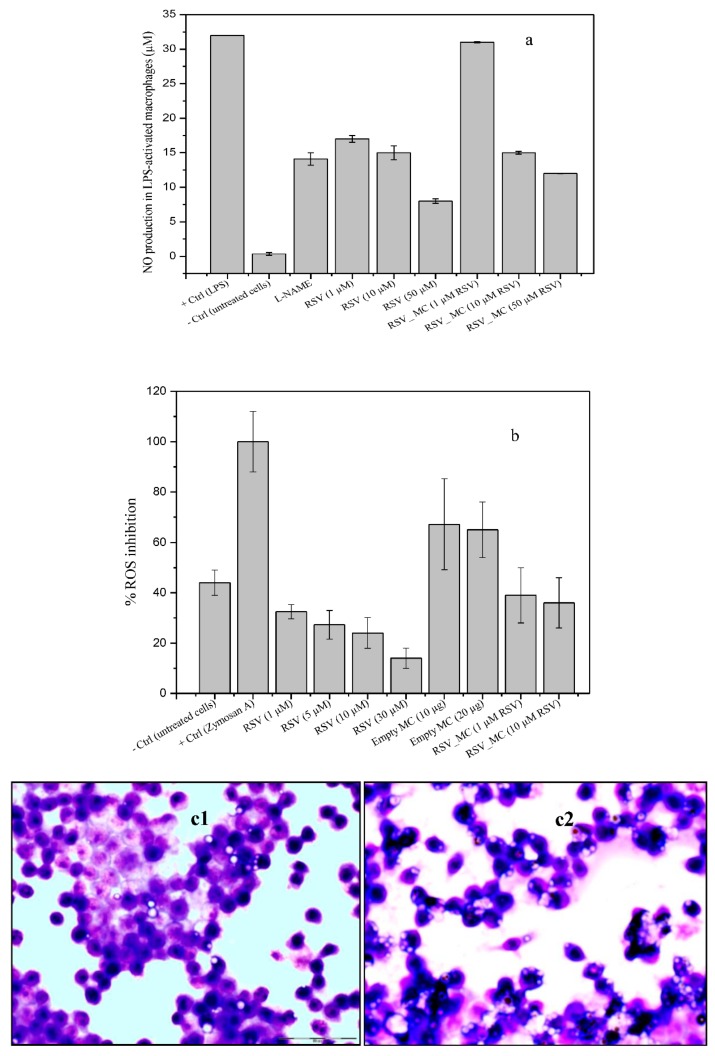

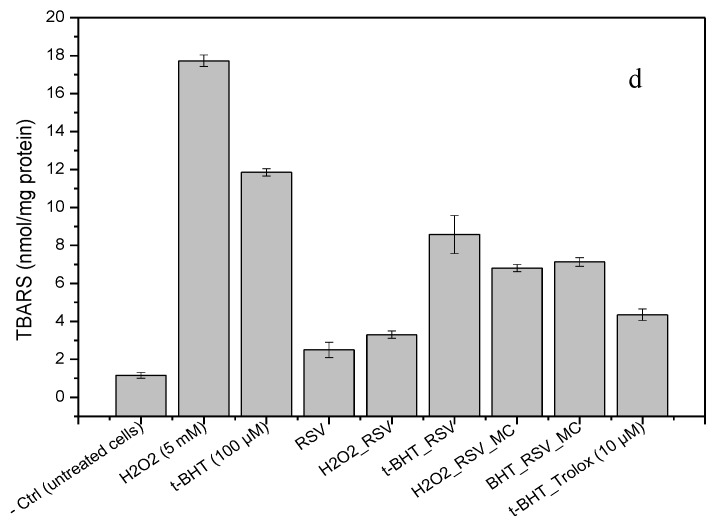
Biological responses of macrophages upon co-incubation with microcapsules: (**a**) Nitrite levels in supernatants of 24 h cultures on RAW macrophages pre-stimulated with LPS (0.5 μg/mL); RSV concentration used for this assays was 10 µM; (**b**) Effect of RSV and RSV-loaded microcapsules on the ROS production after co-incubation with zymosan A-activated macrophages. Phagocytosis assay, (**c1**) blank microcapsules in co-culture with RAW macrophages and (**c2**) RSV-loaded microcapsules (20X). (**d**) Effect of RSV and RSV-loaded microcapsules on H_2_O_2_ and *t*-BHT-induced changes in TBARS levels on RAW macrophages.

In these set of experiments, macrophages were treated with RSV, blank microcapsules, and RSV-loaded microcapsules, for 24 h in the absence or presence of LPS (0.5 µg/mL). The treatment of RAW macrophages with free RSV (10–100 µM) and blank microcapsules had no effect on basal nitrite formation, as measured by the Griess reaction (data not shown). In the following experiments, macrophages were stimulated with LPS. As a control, L-NAME (NO inhibitor, 1 mM) was added to inhibit NO production. As can be seen in Figure 9a, all RSV treatment significantly reduced the NO production, although part of this dose-dependent reduction effect could the possible cytotoxicity of RSV above 50 µM (as the WST-1 assay suggested). This effect was also found when RSV was co-incubated as a microencapsulated form, except for the lowest concentration of microcapsules investigated. Empty microcapsules did not exert any effect on NO production, reaching similar values to the positive control, suggesting the reducing effect on nitrite level reduction was mainly due to encapsulated RSV, either acting from outside or even after they were ingested by the cells. Reduction of nitrite formation after treatment of cells with RSV have been reported previously [42,43,44,45] and even also reduced the expression of cytosolic iNOs protein [37,42,46,47].

### 3.6. ROS Production

ROS, when generated in low concentrations, plays an important role as a signaling molecule in a number of cell process, as follows: Phagocytosis, apoptosis, growth and proliferation, or redox signaling. However, if intracellular reactive oxygen free radical concentrations are increased (overproduction), they can significantly damage cellular components (oxidative stress), or even lead to cell death. Reactive oxygen species are involved in tissue injury in inflammatory diseases when it is generated after phagocytic stimulation. In addition, it is widely accepted that oxidative stress is related to the pathogenesis of the inflammatory joint diseases in patients, for example, rheumatoid arthritis [2,48,49]. Therefore, here we investigated the in vitro response of macrophages upon exposure to RSV-loaded microcapsules. ROS production in response to in vitro stimulation with zymosan A (positive control), RSV, and RSV-loaded microcapsules was examined using DCF-DA (Figure 9b).

In this study, ROS formation and inhibition was expressed as a percentage of fluorescence relative to cells stimulated with zymosan A (positive control). The co-incubation of cells with free RSV or microcapsules alone (empty) or with encapsulated RSV did not shown ROS production, with very similar levels to control cells (basal ROS level). However, cells stimulated with zymosan A produced very high levels of ROS, which were decreased upon the exposure to free RSV in a dose-dependent manner, although the highest concentrations used in these experiments could possibly affect the macrophage viability. Additionally, ROS levels were decreased when empty microcapsules were co-incubated, but this effect was more pronounced in the case of RSV-loaded microcapsules, again, in a dose-dependent manner. For better comparison, in these set of experiments, the amount of RSV in microcapsules was in equal amount to free RSV. Therefore, the higher inhibition of ROS production by free RSV suggested an obvious lesser availability of RSV molecules from loaded microcapsules, from which release is more prolonged. Our results have also shown a small effect of empty microcapsules on ROS decrease generation upon microcapsule uptake (around 25%), relative to the positive control. The reason for this effect remains inconclusive at the moment, although it suggests interference. It should be noted that the microcapsule concentrations used in this experiment were not toxic to the cells.

In summary, in this work, the data on ROS response upon incubation with RSV-loaded microcapsules have shown a significant decrease of cellular ROS by RSV, independent of the macrophage origin [43,50], which confirmed the RSV preservation as an ROS inhibitor on stimulated macrophages when encapsulated.

### 3.7. Phagocytosis Assay

To investigate the phagocytic activity of empty and RSV-loaded microcapsules by macrophages, the percentage of cells ingesting at least one microcapsule was quantified. It could be observed that blank microcapsules showed slightly attenuated phagocytic activities, by 19.3% ± 3.5% compared to 99.06% ± 2.4%, corresponding to RSV-loaded microcapsules engulfment (Figure 9c). In these experiments, no LPS-induced macrophages were tested for phagocytic ability, only naïve ones, so it could be the reason for the low values obtained for phagocytosis. More studies should be required to go more in depth into mechanisms underlying the RSV effect in cells. We hypothesize this behavior could be due to the fact that RSV acts as a modulator of the cell immune response in opposite directions, depending on the doses, with an activating role at low doses or, in contrast, playing an inhibitory role at high doses [51,52,53] Furthermore, more likely, it might also depend on the pathway used by RSV to exert its mechanism of action. In fact, the phagocytosis studies were performed over 4 h, whereas the NO and ROS production assays took 24 h. During the 4 h time used in the phagocytosis study, at least 55% of the loaded amount was released, as release studies have shown. It is reasonable to speculate that it was too small a dose to affect the cell response at that short time, but sufficient to observe some initial signal of activation performance form phagocytosis. 

### 3.8. Lipid Peroxidation in Cell Culture

To determine the impact of protection against lipid oxidation in cell membranes of RAW 264.7 macrophages, pro-oxidant molecules, such as *tert*-butyl hydroperoxide (*tert*-BHT) and H_2_O_2_, have been used as lipid peroxidation inducers. Malondialdehyde (MDA), an aldehyde that is an end-product of lipid peroxidation, has been used as a biomarker of lipid peroxidation through its reaction with thiobarbituric acid (TBA). In the present study, the thiobarbituric acid reacting substances test (TBARS) was used to quantify MDA in cell samples. In an in vivo situation, an excessive MDA production can react with proteins, RNA, and DNA to form adducts. These MDA-induced alterations have been associated with different molecular damages [54] and, further, are highly immunogenic [55]. In our experiments, the oxidant substance used as positive control attacked the polyunsaturated fatty acids that belong to the cell membrane, leading to the impairment of its function (lipid peroxidation). In response to membrane lipid peroxidation level, the cells would promote their survival trough defense mechanisms by upregulating antioxidant proteins or, in contrast, if the extent of the oxidative exceeds the repair capacity of the cells, apoptosis will be induced. Therefore, lipid peroxidation may be used as marker or indicator of damage to cells by free radicals, in rheumatoid arthritis for example [11].

A lipid peroxidation assay performed in macrophages revealed a large content of TBARS in H_2_O_2_ and *t*-BHT–exposed cells (Figure 9d). Incubation with 10 µM of RSV significantly decreased TBARS, suggesting that lipid peroxidation was decreased by RSV treatment. The exposure of cells to RSV-loaded MC (with the same amount of RSV than those used for free RSV experiments) also showed a significant decrease of TBARS levels, although they were higher when compared to RSV or Trolox (a potent antioxidant molecule). The uncontrolled oxidative stress in cells causes direct damage to lipids. The high levels of free radicals or reactive oxygen species (ROS) are produced by the mitochondria, plasma membrane, endoplasmic reticulum, or peroxisomes, upon exposure to different stress conditions or exogenous stimuli, such as infections, toxins, pharmaceutical substances, or formulations. Therefore, free and loaded-RSV seemed to play a role as an inhibitor of lipid peroxidation in cells, at our experimental conditions. However, it must be noted that MDA levels were determined by a TBARS assay, which is known to be strongly affected by the reaction conditions used between laboratories. Therefore, direct comparison to the data provided by the different researchers is often complicated. Even so, these results are in consonance with the inhibition of lipid peroxidation by RSV founded in vivo, explained by various mechanisms [56], and also in cell cultures [56,57].

## 4. Conclusions

The present work has successfully established the preparation of RSV-loaded microcapsules made of PCL by ultrasonic atomization, with a size and morphology suitable for local administration into the joint by intra-articular administration. The stability and antioxidant properties of encapsulated RSV were preserved by microencapsulation. The microcapsules showed no cytotoxicity and have demonstrated an enhanced efficacy to control signals of inflammation and oxidative stress in LPS-activated mouse macrophages, a classical feature of inflammatory disorders affecting the joint.

Further studies should be conducted in the future to explore the clinical significance of the developed formulations based on RSV encapsulated in PCL and, more specifically, the RSV dose normalization. Although the biological relevance of RSV could differ greatly from the drugs used in inflammatory therapies for treating joint disease, the results presented here, using encapsulated RSV in mouse macrophages, is expected to have an anti-inflammatory effect that may be useful in retarding the progression of joint diseases, alone or in combination with the usual drugs used in these therapies. In addition, the results of this study provide additional support for developing microcapsules intended to intra-articular administration to the synovial cavity and the remaining effective concentrations of the drug for prolonged times. Future animal studies are required to investigate the potential benefits of site-specific administration and, subsequently, the reduction of dosage frequency, and to increase the availability of antioxidant molecules in inflamed joints.

## Figures and Tables

**Table 1:** Process parameters investigated in RSV-loaded microcapsules formulations (the inner and outer channel flow were both 3 mL/min), yield of the formulation process (%), and encapsulation efficiency (E.E) for every RSV formulation.

Formulation	[PCL]	Power	[PVA]	Process Yield	Encapsulation Efficiency (E.E)
F1	0.08%	5 W	3.5%	85.83%	39.66%
F2	0.5%	4 W	3.5%	85.45%	63.80%
F3	0.5%	6 W	3.5%	80.43%	50.20%
F4	1.5%	3.58 W	3.5%	85.30%	83.97%
F5	1.5%	5 W	3.5%	92.07%	96.00%
F5	1.5%	5 W	3.5%	92.07%	96.00%
F5	1.5%	5 W	3.5%	92.07%	96.00%
F5	1.5%	5 W	3.5%	92.07%	96.00%
F5	1.5%	5 W	3.5%	92.07%	96.00%
F6	1.5%	6.41 W	3.5%	80.22%	66.16%
F7	2.5%	4 W	3.5%	86.12%	77.30%
F8	2.5%	6 W	3.5%	89.88%	91.48%
F9	2.91%	5 W	3.5%	91.88%	92.76%
